# Age-Matched Comparative Analysis of Binocular Vision Anomalies among Children with Dyslexia in Northern Nigeria

**DOI:** 10.3390/pediatric16030048

**Published:** 2024-07-15

**Authors:** Ismail Salma Mukhtar, Ngozika Esther Ezinne, Mizhanim Mohamad Shahimin, Bariah Mohd-Ali, Eki Oghre, Ferial M. Zeried, Uchechukwu Levi Osuagwu

**Affiliations:** 1 Optometry and Vision Science Program, Centre for Community Health Studies (ReaCH), Faculty of Health Sciences, Universiti Kebangsaan Malaysia, Kuala Lumpur 50300, Malaysia; p112690@siswa.ukm.edu.my (I.S.M.); mizhanim@ukm.edu.my (M.M.S.); bariah@ukm.edu.my (B.M.-A.); 2 Department of Optometry, Bayero University, Kano 700241, Nigeria; 3 Bathurst Rural Clinical School (BRCS), School of Medicine, Western Sydney University, P.O. Box 9008, Bathurst, NSW 2795, Australia; ezinne.ngozi@gmail.com; 4 Optometry Unit, Department of Clinical Surgical Sciences, University of the West Indies, Saint Augustine Campus, St. Augustine 685509, Trinidad and Tobago; 5 Department of Optometry, University of Benin, Uselu, Benin City 300103, Nigeria; eki.oghre@uniben.edu; 6 Department of Optometry & Vision Sciences, College of Applied Medical Sciences, King Saud University, Ilesha 2915, Saudi Arabia; drferyal@ksu.edu.sa

**Keywords:** dyslexia, binocular vision, visual symptoms, children, Nigeria

## Abstract

**Background**: Dyslexia, a neurodevelopmental disorder affecting reading skills, poses significant challenges to children’s academic performance and quality of life. Despite its rising prevalence and adverse effects, understanding of its relationship with vision anomalies remains limited, particularly in low-resource settings like Nigeria. This study aims to assess the prevalence of binocular vision anomalies (BVAs) among children with and without dyslexia in Kano, Nigeria. **Methods**: This is a hospital-based, cross-sectional, matched-paired, controlled study conducted at the Aminu Kano Teaching Hospital (AKTH) Eye Clinic in Northern Nigeria. The study included school children who visited the AKTH Eye Clinic from January 2018 to December 2022. Visual acuity tests, external eye examinations and accommodative, binocular vision and oculomotor skills tests were conducted. Descriptive statistics, independent *t*-tests, Mann–Whitney U tests and Fisher’s exact tests were conducted, with a significance level set at *p* < 0.05. **Results**: Forty-four children aged 12 ± 2 years participated. Children with dyslexia reported higher rates of visual symptoms than those without dyslexia, Blurring vision, visual distortion and eye strain were the most prevalent (*p* < 0.05) BV symptoms. Accommodative insufficiency), was the most common visual abnormality, and was significantly higher in children with dyslexia than those without dyslexia (45.5% vs. 18.2%). However, other visual anomalies showed no significant difference between groups. There was a high prevalence of binocular vision anomalies in both groups. Binocular test findings showed dyslexic children had significantly lower distance positive fusional vergence recovery values (*p* = 0.005). All cases of convergence insufficiency alone were found in the non-dyslexic group. **Conclusions**: The study found that children with dyslexia residing in Northern Nigeria demonstrated higher rates of visual symptoms, more accommodative insufficiency and lower distance positive fusional vergence recovery values compared to their non-dyslexic counterparts.

## 1. Introduction

Dyslexia is a neurodevelopmental disorder characterized by difficulties in phonological skills including the ability to read, write, spell and speak, and this poses significant challenges to children’s academic performance and quality of life [1,2]. Despite its impact, dyslexia frequently goes undetected, and with prevalence rates of 5% to 17.5% in children, more than two-thirds of children with a learning disability have dyslexia, which is the most common learning disability among children [1,3]. While dyslexia is well documented globally, there remains a gap in understanding the relationship between dyslexia and visual anomalies, particularly in low-resource countries like Nigeria. Dyslexia can negatively impact quality of life, social skills, academic performance and self-esteem in children if not identified on time [4,5,6]. Previous studies carried out on children with dyslexia when compared to their control counterparts have shown dysfunctions in areas of accommodation (accommodative convergence/accommodation ratio, amplitude accommodation) and vergence (convergence insufficiency) [7,8,9,10]. However, there is a notable absence of research specifically addressing binocular vision anomalies (BVAs) among dyslexic children in Nigeria.

The prevalence of visual anomalies among dyslexic children has been assessed in some parts of the world, and its variation ranges from high to low [8]; in the USA, it was found to be very high, with a prevalence of 79% [11], and in Austria, the prevalence was estimated to be 33.9% [8], while there is also a report of a lower prevalence of less than 20% [11]. In Africa, there is only one study from South Africa conducted more than a decade ago, showing that accommodative infacility was prevalent among people with dyslexia [12]. There is a need to present recent data on dyslexia in Africa. The city of Kano in Nigeria was chosen for this study for several reasons. Firstly, Kano’s nascent optometry profession and lack of optometric-related studies make it an ideal setting for pioneering research. Additionally, the study’s aim to elucidate the relationship between dyslexia and BVA in Kano fills a critical gap in the literature, providing valuable insights for clinicians and policymakers. Kano’s diverse population, robust healthcare infrastructure, and cultural richness offer unique perspectives on dyslexia’s prevalence and its impact on the community. Collaborative efforts with local stakeholders further enhance the research’s relevance and ensure the effective translation of findings into actionable interventions. Therefore, choosing Kano as the study location is not only strategic but also imperative for addressing the challenges dyslexia poses in this underserved region. This study aims to assess and compare the prevalence of BVA among children with and without dyslexia in Nigeria; thus, it is a hospital-based study that provides essential information to guide strategic optometry interventions for clinicians and public health policymakers.

## 2. Methodology

### 2.1. Study Design and Setting

This is a cross-sectional, matched-paired, controlled study conducted at the Aminu Kano Teaching Hospital (AKTH) Eye Clinic, in Northern Nigeria. AKTH is a Federal Government Teaching Hospital located in Kano State, Nigeria. It was formerly known as Bayero University Teaching Hospital. It doubles as both a medical and tertiary center for training medical students and resident doctors. The participants were school children who visited the AKTH from January 2018 to December 2022 with and without dyslexia.

### 2.2. Ethics

This research project was registered in Universiti Kebangsaan Malaysia (UKM) and was approved by the Research and Ethics Committee of UKM: UKM.FSK.PNI.800-2/2/1 (JEP-2023-158). Permission to access the patients’ files in Nigeria was obtained from the chief medical director of the Aminu Kano Teaching Hospital (AKTH), Nigeria. Information and reasons to participate in the study were explained to the parents and caregivers of the children. Written consent was signed by all parents, and assent was obtained from all children before data collection. The study was conducted according to the Helsinki Declaration about the use of human subjects in research studies.

### 2.3. Sample Size and Sampling Technique

G*power software version 3.1.9.7 [13] was used to determine the minimum sample size using data from a similar study conducted in South Africa [12]. A calculated sample size of 22 children in each group was determined for a direct group comparison sufficient to achieve 80% statistical power with an attrition rate of 15% at a 95% confidence interval. Purposive sampling of children diagnosed with dyslexia and age-matched healthy controls without dyslexia was conducted to recruit from children attending the hospital to compare the findings.

### 2.4. Inclusion and Exclusion Criteria

Children diagnosed with dyslexia by an educational psychologist at the eye clinic during the study period were eligible to participate in the study. Children with no specific history of dyslexia, no history of speech therapy, and no intellectual disability in reading skills were included in the control group. Children were excluded if they had undergone dyslexia intervention or had a history of mental or neurological disorders or conditions that affect binocular vision.

### 2.5. Procedure

This study was conducted in a hospital and the case files of children who visited the hospital within the study period and were diagnosed with dyslexia were requested from the hospital administrator. A matching control group was conveniently selected from the hospital records. The parents of the children were contacted over the phone and invited to participate in the study. Information about the study and reasons to participate in the study were explained to them, and those that gave their consent to participate in the study were advised to bring their children. All procedures to be conducted were explained to the children and their parents. Consent was obtained from all children where necessary before data collection. A comprehensive routine eye examination was carried out in a standard clinic cubicle under room illumination, and the children underwent a comprehensive accommodation and binocular vision (BV) assessment. Details of these examinations and how they were performed are presented in Table 1.

Ocular *symptoms* Ocular symptom: A questionnaire was used to determine the ocular and non-ocular signs and symptoms of all children. The questionnaire was developed from similar studies and validated; first, it was pilot-tested on a group of people, and based on their responses, the questionnaire was modified. Questions regarding their near work were asked, and all responses were recorded. The ocular symptoms were eyestrain; visual distortions while reading; blurring vision; frequent blinking; asthenopia; and others.

Non-ocular symptoms: Each child was asked about non-ocular symptoms, and their responses were noted. The non-ocular symptoms were letter and word recogni-tion; understanding words and ideas; low reading speed and fluency; and general vocabulary skills.

#### Tests

External eye examination External eye examination was performed using the slit lamp biomicroscope to examine the external structures of the eyes for any abnormality.

Visual acuity was assessed using the Snellen chart at 6 m for distance vision and at 40 cm for near visual acuity. The measurement was carried out monocularly and then binocularly [1].

The refractive status of all patients was determined with the cycloplegic and subjective refractions. Cycloplegic refraction was carried out using 1% Tropicamide and cyclopentolate; two drops of each drug were given at an interval of 5 min each. The refractive status was checked by scoping both meridians and neutralizing any movement found using a retinoscope. Subjective refraction was carried out with a phoropter by determining the maximum spherical plus and the least minus lens corrections that gave the best visual acuity. Types of refractive error were determined by the spherical equivalent (SE) power. Myopia was defined as SE ≤ −0.50 D, hyperopia as SE ≥ +1.00 D, and astigmatism as SE ≥ −0.75. Amblyopia (lazy eye) was defined when visual acuity was ≤20/30 or there were two-line intraocular optotype acuity differences with no pathology, after the correction of the refractive error [1].

Accommodation status was determined by measuring the amplitude of accommodation (AA), dynamic retinoscopy, and accommodative facility (AF). AA was performed using Donder’s push-up-to-blur method with a target of one line above the patient’s best near visual acuity. An accommodative target was brought closer to the eyes until a sustained blur was reported. The distance was measured in cm and converted to diopters by dividing by 100 (AA). This was carried out monocularly and binocularly [15].

Accommodative response or dynamic retinoscopy was carried out using the monocular estimation method (MEM). The MEM card was attached to the retinoscope and the child was asked to read the letters on the MEM card using both eyes while the horizontal meridian was scoped until movement was fully neutralized by plus lenses. The amount of lens used indicates the lag or lead of accommodation [15].

The accommodative facility was tested using ±2.00D flippers and a near card at 40 cm. The child reported clear once the letters were clear for one minute. The number of flips for clearing the plus and minus flippers made a complete cycle. The number of cycles was recorded per minute, and was carried out binocularly and then monocularly as described previously [16].

Accommodative insufficiency was defined as a reduced amplitude of accommodation than expected for the patient’s age using Hofstetter’s calculation for minimum amplitude: 15–0.25 (age). This was accompanied by difficulty with the ±2.00 D flipper test and positive relative accommodation (PRA) under −1.50 D. Accommodative infacility was defined as difficulty with both plus and minus lenses in monocular accommodative facility testing with <7 cycles per minute (cpm) for 8–12 years old and <13 cpm for 13 years and above with ±2.00 DS and difficulty with both plus and minus lenses in binocular accommodative facility testing with <5 cpm using ±2.00 DS.

Binocular vision test: Phoria or tropia was performed with the cover test. With the patient eyes in the primary position, the examiner occluded and un-occluded the eye while observing for movement in the uncovered eye. When strabismus was found, no other binocular test was subsequently carried out [8,14].

Vergence status was determined by performing the near point of convergence (NPC), and the positive and negative fusional vergence (PFV/NFV) tests were carried out using a push-up technique with a pencil tip as a target until the child reported a break. This was repeated three times, and the average reading was taken [15,17]. PFV/NFV tests were carried out using prism bars to assess the fusional vergence amplitude and recovery. The prism bar was placed in front of the patient’s eye (base in for NFV and base out for PFV) and increased continuously from zero until the patient reported the target to be blurred, broken, and recovered for distance and near distances; base in was assessed before base out because of the convergence effect. Subsequently, a vertical prism was placed in front of the eye in a base up and down direction at a time until reports of break and reduced until reports of recovery. All tests were carried out with subjective refraction in place [18]. Fusional vergence dysfunction is defined as when near NFV break < 12 BI, near PFV break < 23 BO, distance NFV break < 7 BI and distance PFV break < 11 BO. Table 2 presents the criteria for the diagnosis of the performed tests.

Oculomotor testing was performed using the developmental eye movement (DEM) test. The DEM test is a paper-based oculomotor test aimed at giving an indirect measure of eye movement in a reading-like condition. The DEM test has three subtests: a pre-test, a vertical subtest and a horizontal test. The vertical subtest depends on the individual’s visual verbal automatic skills. The horizontal subtest consists of numbers arranged in a non-symmetrical horizontal array that assesses the horizontal saccadic function. This was carried out to quantify eye movement while reading; the subject was asked to read the pre-test, the vertical test and the horizontal test. The time and error were noted for the two sub-groups. The horizontal time score was determined by adjusting the time to complete test C by compensating for errors. The ratio score was calculated by dividing the adjusted horizontal time by the vertical time [19].

Binocular vision anomalies were categorized based on the binocular vision tests performed. For diagnosis purposes, a minimum of two signs from the parameters detailed in Table 2 were used [17].

Convergence insufficiency is defined as exophoria > 4 BI greater for near than distance, NPC > 6 cm break with an accommodative target, and PFV < 15 BO (break or blur value). Divergence insufficiency is defined as esophoria > 2 BO for distance and NFV < 7 BI (break–step vergence for distance). Convergence excess is defined as significant esophoria at near, >2 prisms, reduced negative fusional vergence < 12/10 for break and recovery, and high MEM, >+0.75 DS. Divergence excess is defined as intermittent to constant exodeviation at a distance that is 5 PD greater than at near, and a low PFV break value of <11 BO at distance.

### 2.6. Data Analysis

The data collected were imported to the Excel spreadsheet and exported to the Statistical Package for Social Sciences (SPSS) version 25.0 (Armonk, NY, USA: IBM Corp.). Data were presented using proportions and descriptive analysis was performed using independent *t*-test, and Mann–Whitney U test to compare values of quantitative BV parameters between groups. Categorical data were analyzed using Fisher’s exact test including comparison of the types of accommodative and vergence anomalies between the two groups. The significance level was set at *p* < 0.05.

## 3. Results

### 3.1. Demographic Characteristics of the Study Participants

A total of 44 children with a mean age of 12 ± 2 years participated in the study. About half (54.5%) were male and three-quarters were from high socioeconomic status (79.5%). Compared with the control group, a higher proportion of the children with dyslexia had a low socioeconomic background (31.8% vs. 9.1%, *p* = 0.07). Visual acuity and refraction were similar between the groups, irrespective of the tested eye. However, the blurring of vision, visual distortion, and eye strain were prevalent among the children with dyslexia, and they were statistically significant (*p* < 0.05) (Table 3).

### 3.2. Binocular Vision Test Findings

Table 4 presents the mean differences in all the binocular vision test findings conducted for dyslexic and non-dyslexic children. the results of independent *t*-test and Mann–Whitney U test comparing the average values of accommodative and BV parameters between dyslexic and non-dyslexic children are also shown.

The summarized data showed that all accommodative tests, which included AA, DR and AF, did not attain statistically significant differences, though they appeared slightly lower in the dyslexic than in non-dyslexic participants (*p* > 0.05) (Table 4). The phoria test near and far did not attain a statistically significant difference with a mean ± SD of 10 ± 3.3 vs. 7 ± 3.2 (*p* > 0.70) and distance of 2.0 ± 1.68 vs. 2.0 ± 1.26 (*p* > 1.00). The values of stereo acuity and NPC were markedly skewed. Median (IQR) values for stereo acuity were 45 ± 2.24 s of arc in dyslexic and 40 ± 2.25 s of arc in non-dyslexic children. The NPC was on average about 5 cm higher in dyslexic children (10.7 cm) than in non-dyslexic children (5.8 cm).

In the dyslexic group, the mean NFV (DNFV) break and recovery value at distance was slightly lower than that of the non-dyslexia group, (10.2 ± 3.9 ΔD vs 2.8 ± 3.4 ΔD respectively), but this was not statistically significant. The distance PFV (DPFV) recov-ery value was significantly different between the two groups with a mean value of 4.6 ± 3.9 ΔD vs. 8.6 ± 5.9 ΔD (*p* = 0.005) for the dyslexic children and non-dyslexic groups, respectively.

The mean DEM value for the horizontal test was on average statistically signifi-cantly higher (74.3 ± 44.9 vs. 51.9 ± 15.9, *p* = 0.04) in the in dyslexic than the non-dyslexic group. However, the vertical DEM and DEM ratio was similar between the groups (*p* > 0.05) (Table 4).

### 3.3. Prevalence of Accommodative and Binocular Vision Anomalies

**Figure 1:**
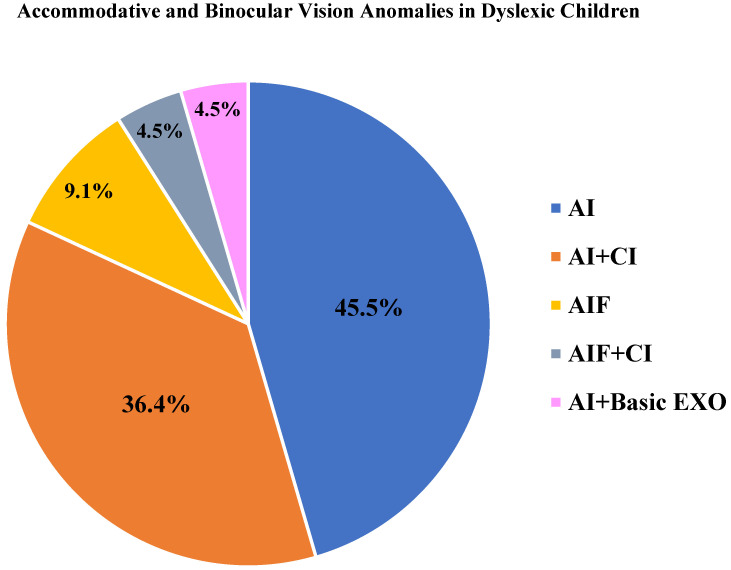
Accommodative and binocular vision anomalies in dyslexic children. AI: accommodative insufficiency, AI + CI: accommodative insufficiency + convergence insufficiency, AIF: accommodative infacility, AIF + CI: accommodative infacility + convergence insufficiency, AI + Basic EXO: accommodative insufficiency + basic exophoria.

All children with dyslexia (n = 20) and 90% of those without dyslexia in this study had BVAs (*p* = 0.49). Figure 1 demonstrates the distribution of accommodative and BV functions in the dyslexic group. Accommodative insufficiency was the most prevalent visual anomaly, followed by a combination of accommodative insufficiency with convergence insufficiency (*p* > 0.05). Accommodative insufficiency was significantly more prevalent in children with dyslexia compared to their counterparts (45.5%) (Figure 1). Cases of Accommodative insufficiency + convergence insufficiency were similar between groups (36.4% vs 27.3%, *p* = 0.52).

#### 3.3.1. Accommodative and Binocular Vision Anomalies in Non-Dyslexic Children

**Figure 2:**
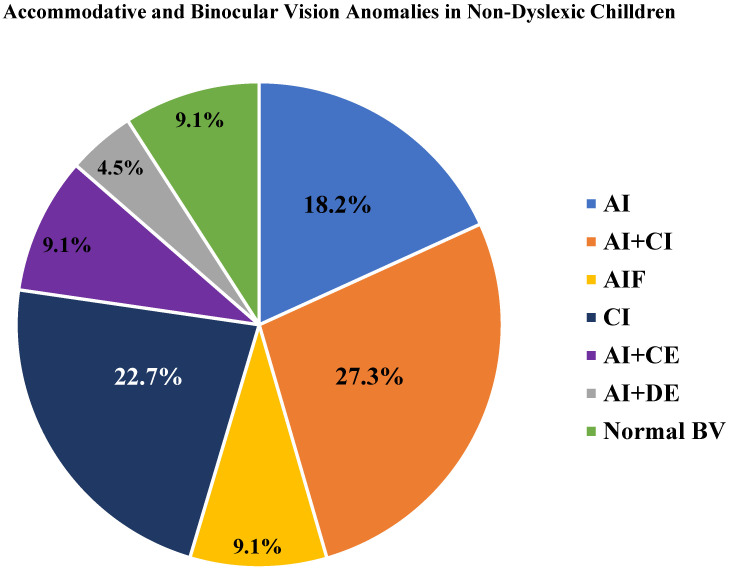
Accommodative and binocular vision anomalies in non-dyslexic children. AI: accommodative insufficiency, AI + CI: accommodative insufficiency + convergence insufficiency, AIF: accommodative infacility, CI: convergence insufficiency, AI + CE: accommodative insufficiency + convergence excess, AI + DE: accommodative insufficiency + divergence excess, Normal BV: normal binocular vision.

Figure 2 demonstrates accommodative and BVAs in non-dyslexic children; 27.3% of the dyslexic group had similar Accommodative insufficiency + CI values compared to the controls (*p* = 0.52), followed by convergence insufficiency alone (CI). All cases were found only in the non-dyslexic group (22.7%). Although 9.1% of children in the non-dyslexic group had normal BV these variables were similar when compared with the non-dyslexic group.

#### 3.3.2. Distribution of Accommodation Insufficiency

**Figure 3:**
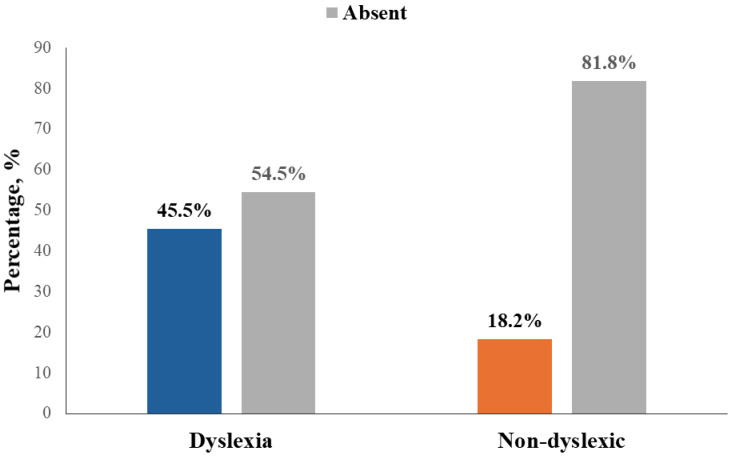
Distribution of Accommodative insufficiency in the dyslexic and non-dyslexic groups.

Figure 3 demonstrates the distribution of accommodative insufficiency in dyslexic and non-dyslexic children; AI was more common in the dyslexic group, (45.5% versus 18.2%) with approximately 54.5% of the children in the dyslexic group reporting the absence of AI as compared to fewer children in the non-dyslexic group.

## 4. Discussion

Our study was the first to compare the prevalence of binocular vision anomalies in dyslexic and non-dyslexic children in Nigeria. Symptoms of BVA including eye strain, blurring of vision, and visual distortion were significantly more common among children with dyslexia. Overall, AI was significantly more common among dyslexic children. Visual anomalies were prevalent in both groups. AI and CI were the most prevalent anomalies among the two groups. Although both groups had a reduced recovery time of fusional vergence, it was significantly lower among the children with dyslexia.

Dysfunction in the visual magnocellular area was reported to have a great impact on visual function, including a slow response to accommodation in dyslexic children [11,20]. This is consistent with the high prevalence of AI recorded in the current study and other studies [8,10,11,21]. Also, a high prevalence of accommodation infacility was recorded among dyslexic children in South Africa and Canada [7,12]. Poor accommodation ability would limit reading and focusing ability in children, which could lead to poor academic performance in children [22,23,24].

Magnocellular dysfunction or cortical hyperexcitability in children with dyslexia, known to induce visual stress, could be the reason for the high level of BVA symptoms recorded in children with dyslexia in our study. This finding was consistent with a previous report [11,25], suggesting the need to check for BVA symptoms in dyslexic children for early detection and management. Contrary to the present study, [26] revealed that phonological deficits may be the cause of symptoms seen in reading in children with dyslexia, and subsequent training of phonological skills improved their reading.

Various studies [10,11,27,28,29] have reported a higher prevalence of BVA in children with dyslexia compared to those without dyslexia. This is similar to our result, though slightly higher than others. The difference in the findings could be attributed to methodological variations between our study and the previous studies, such as the inclusion of only children in our study versus both children and adults in a previous study [10].

Also, variations in age, visual acuity and the refractive status of the control group in our study could be another reason for the variations. For example, the mean age of the control group in our study was lower than the 14.25 ± 1.67 years reported in a similar study in Sweden [25]. Also, the refractive status of our control group was higher than what was recorded in a study in Sweden [25].

Convergence insufficiency was implicated as one of the abnormalities found in dyslexic children in numerous studies in Finland [30], Austria [8], Iran [31] and Brazil [28]. This could be due to binocular instability commonly found among children with dyslexia. Convergence Insufficiency Treatment Trial—Attention & Reading Trial (CITT-ART) findings also revealed a high prevalence of CI in children, especially during near work or reading [32]. In contrast, CI was observed to be more prevalent among the non-dyslexic group in the current study. Also, other studies [32,33] recorded no significant difference in the prevalence. The high prevalence of CI recorded in our study among the control group could be due to there being more children with myopia in the control group, and CI is usually common in myopic children. It is worth noting that most of the children screened into the study were without optimum refractive correction. Subsequent tests were carried out using their optimally corrected subjective refraction, but this alone cannot correct the existing symptoms and signs of their binocular vision disorder. In addition, our study did not assess CI symptoms with the Convergence Insufficiency Symptom Survey (CISS) since CI symptoms present in some children without signs. A further study that will assess the prevalence of CI symptoms in dyslexic children using the CISS is highly advised.

Similar to the present study, previous studies [7,24,28,29,34] have found significant differences in the recovery time among dyslexic children. Dyslexic children tend to have poor negative fusional reserve (NFV) due to poor fixation and tracking ability, resulting in reduced recovery time. This could be the case in our study. The need for visual skills training for dyslexic children is highly advised to help reduce the burden of dyslexia on children with or without the use of special educational training or visual aids [22,23,35].

## 5. Limitations and Recommendations

Our study has several limitations that should be considered when interpreting the results. The study prevalence was based on using hospital patients; hence, the findings cannot be generalized for the entire population because those who did not visit the clinic were not included. The sample size was relatively small for this representation. Although the sample size calculation justified valid results, future research should aim for a larger sample size to enhance generalizability. Participants were recruited from a single center, which may restrict the applicability of our results. Broader recruitment across multiple centers would strengthen the validity of our conclusions. Another limitation was that the study did not explore cognitive skills (reading) or additional visual functions such as contrast sensitivity and color vision in dyslexia. Investigating these aspects in future studies would provide a more comprehensive understanding. Despite these limitations, our study provides valuable insights into the prevalence of BVA within specific groups, allowing for meaningful comparisons with global data.

## 6. Conclusions

Accommodation and convergence insufficiencies were the most prevalent visual anomalies in dyslexic children. There was a high prevalence of BVA among children in Nigeria, especially those with dyslexia. Nigerian children with dyslexia had higher rates of visual symptoms and lower distance positive fusional vergence recovery values compared to their non-dyslexic counterparts. These findings underscore the importance of routine binocular vision assessments and regular vision therapy for children with dyslexia to optimize their academic performance and quality of life. Integrating interventions targeting BVA into management plans and implementing comprehensive vision screenings in school health programs can enhance outcomes for children with dyslexia, particularly in low-resource settings. These measures will help in reducing the burden of visual problems associated with dyslexia.

## Figures and Tables

**Table 1:** Normal values of tests [14].

Parameters	Technique	Normal Range
Distance VA (visual acuity)	Snellen chart	6/9 or better
Near VA	Snellen chart	N6 or better
Subjective refraction		≤+0.50 DS and ≤−0.50 DS
(a) Accommodation test		
Amplitude of accommodation	Donder’s Push up	2.00DS less than calculated Hofstetter’s formula
Dynamic retinoscopy	MEM	plano to +0.75
Accommodation facility	±2.00 flippers test	7CPM < 12 years 11CPM > 13 years OU: 5CPM < 12 years 10CPM > 13 years
(b) Binocular vision test		
Stereoacuity	Titmus fly	40 sec of arc
Phoria test	Cover test	Near 0–6 exophoria Distance 0–2 exophoria
NPC	Push up	5 cm
Distance Negative Fusional (BI) break/recovery	Prism bars	5/3
Table continued Near Negative Fusional (BI) blur/break/recovery	Prism bars	11/19/10
Distance Positive Fusional (BO) blur/break/recovery	Prism bars	7/15/8
Near Positive Fusional (BO) blur/break/recovery	Prism bar	14/18/7
(c) Oculomotor status		
Oculomotor movement	DEM	Normal
Ocular motor test	Corneal reflex	Symmetric
Saccade test	Alternating between two targets	Accurate
Ocular motility	Broad H	Unrestricted

**Table 2:** Criteria for classification of BVA based on Barrett and Cooper [14,17].

BVA	Criteria
Convergence insufficiency	Reduced AA, reduced NPC (>6 cm), reduced positive fusional range at near (<18, 7) and exophoria greater at near (>4 BI) than at distance.
Convergence excess	Esophoria greater at near (>2 BO), reduced negative fusional range at near (<19, 20) or does not meet the Sheard’s criteria, high MEM (>+0.75 D) and normal NPC
Divergence insufficiency	Esophoria greater at distance (>2 BO) and normal esophoria at near, negative fusional range distance (<5, 3) or do not meet Sheard’s criteria.
Divergence excess	Exophoria is greater at distance than exophoria at near (>2BI), reduced positive fusional vergence at a distance (<15, 8) and normal NPC.
Accommodative insufficiency	Low amplitude of accommodation, low accommodative facility, high MEM (>+0.75 D)
Accommodation excess	Esophoria (>2 BO), lead of accommodation (<+0.25 D), low NPC (>6 cm), low accommodation facility.
Accommodative infacility	Normal amplitude of accommodation, low accommodation facility.

**Table 3:** Demographical characteristics of participants with dyslexia (n = 22) and control (n = 22).

Variables	Dyslexic Group	Control Group	*p*-Value
**Gender**			
Male	12 (54.5)	12 (54.5)	1.00
Female	10 (45.5)	10 (45.5)	
**Age**			
<12 years	10 (45.5)	10 (45.5)	1.00
≥12 years	12 (54.5)	12 (54.5)	
**Child’s educational level**			
Primary	6 (27.3)	7 (1.8)	0.74
Secondary	16 (72.7)	15 (68.2)	
**Parent’s socioeconomic status**			
High	15 (68.2)	20 (90.9)	0.07
Low	7 (31.8)	2 (9.1)	0.74
**Visual acuity**			
Near logMAR (mean ± SD)	RE:3.8 (2.2)	3.1 (1.6)	0.23
	LE: 3.6 (1.9)	3.0 (1.7)	0.33
Distance logMAR (mean ± SD)	RE: 2.9 (1.3)	RE: 3.0 (1.7)	0.84
	LE: 2.8 (1.1)	2.8 (1.8)	0.92
**Refraction, D**			
Subjective refraction (mean ± SD)	RE: 0.33 ± 0.49 LE: 0.33 ± 0.49	RE: 0.24 ± 0.82 LE: 0.17 ± 0.73	0.66
Hyperopia, **n** (%)	5 (22.7)/5 (22.7)	8 (36.4)/8 (36.4)	0.40
Myopia, **n** (%)	0	3 (13.6)/2 (9.1)	
Emmetropia, **n** (%)	17 (77.3)	11 (50.0)/12 (54.5)	
**Ocular signs and symptoms**			
**Eyestrain**	15 (75.0)	3 (18.8)	0.001
**Visual distortion**	15 (68.2)	7 (33.3)	0.02
**Blurring vision**	21 (95.5)	11 (50.0)	0.001
**Frequent blinking**	11 (57.9)	7 (46.7)	0.51
**Asthenopic symptoms**	10 (52.6)	5 (33.3)	0.26

**Table 4:** Binocular vision test findings.

Variables	Dyslexic Group (Mean ± SD)	Control Group (Mean ± SD)	*p*-Value
**Accommodation**			
AA, OD (D)	8.1 ± 3.7	8.9 ± 3.4	0.41
AA, OS (D)	7.9 ± 4.2	9.1 ± 4.2	0.33
AA, OU (D)	8.7 ± 3.9	9.5 ± 4.2	0.55
MEM OD, (D)	1.51 ± 0.68	1.48 ± 0.62	0.86
MEM OS, (D)	1.64 ± 0.69	1.56 ± 0.56	0.68
AF, OD (CPM)	4.4 ± 2.4	4.5 ± 2.9	0.82
AF, OS (CPM)	4.7 ± 2.6	5.3 ± 3.5	0.56
AF, OU (CPM)	4.8 ± 2.7	4.7 ± 3.4	0.92
**Binocular vision**			
Stereopsis (s of arc)	45 ± 2.24	40 ± 2.25	0.67
Phoria near	10 ± 3.3	7 ± 3.2	0.70
Phoria far	2.0 ± 1.68	2.0 ± 1.26	1.00
NPC (cm)	13.6 ± 8.5	10.1 ± 5.8	0.20
Near NFV Break (ΔD)	10.2 ± 3.9	11.8 ± 5.0	0.22
Near NFV Recovery (ΔD)	11.0 ± 5.3	10.2 ± 5.5	0.93
Near PFV Break (ΔD)	18.1 ± 5.8	21.4 ± 7.4	0.10
Near PFV Recovery (ΔD)	6.6 ± 5.3	10.0 ± 6.1	0.06
Distance NFV Break (ΔD)	10.2 ± 3.9	11.8 ± 5.0	0.22
Distance NFV Recovery (ΔD)	2.8 ± 3.4	3.4 ± 4.9	0.71
Distance PFV Break (ΔD)	17.3 ± 5.4	20.1 ± 8.4	0.36
Distance PFV Recovery (ΔD)	4.6 ± 3.9	8.6 ± 5.9	0.005 *
**Oculomotor skill**			
DEM Vertical	74.8 ± 37.2	59.5 ± 18.1	0.27
DEM Horizontal	74.3 ± 44.9	51.9 ± 15.9	0.04 *
DEM Ratio	0.97 ± 0.14	0.89 ± 0.17	0.13

AA: amplitude of accommodation, AF: accommodation facility, MEM: monocular estimation method NPC: near point of convergence, NFV: negative fusional vergence, PFV: positive fusional vergence, DEM: developmental eye movement test. *: show significant *p*-values.

## Data Availability

The data set is available on a reasonable request from the corresponding author.
